# The Stumblemeter: Design and Validation of a System That Detects and Classifies Stumbles during Gait

**DOI:** 10.3390/s21196636

**Published:** 2021-10-06

**Authors:** Dylan den Hartog, Jaap Harlaar, Gerwin Smit

**Affiliations:** 1 Department of Biomechanical Engineering, Delft University of Technology, 2628 CD Delft, The Netherlands; Dylan.den.Hartog@hotmail.com (D.d.H.); G.Smit@tudelft.nl (G.S.); 2 Department Orthopedics & Sports Medicine, Erasmus Medical Center, 3015 GD Rotterdam, The Netherlands

**Keywords:** stumbling, detection, machine learning, inertial measurement unit, accelerometer, gyroscope, amputee, osseointegration

## Abstract

Stumbling during gait is commonly encountered in patients who suffer from mild to serious walking problems, e.g., after stroke, in osteoarthritis, or amputees using a lower leg prosthesis. Instead of self-reporting, an objective assessment of the number of stumbles in daily life would inform clinicians more accurately and enable the evaluation of treatments that aim to achieve a safer walking pattern. An easy-to-use wearable might fulfill this need. The goal of the present study was to investigate whether a single inertial measurement unit (IMU) placed at the shank and machine learning algorithms could be used to detect and classify stumbling events in a dataset comprising of a wide variety of daily movements. Ten healthy test subjects were deliberately tripped by an unexpected and unseen obstacle while walking on a treadmill. The subjects stumbled a total of 276 times, both using an elevating recovery strategy and a lowering recovery strategy. Subjects also performed multiple Activities of Daily Living. During data processing, an event-defined window segmentation technique was used to trace high peaks in acceleration that could potentially be stumbles. In the reduced dataset, time windows were labelled with the aid of video annotation. Subsequently, discriminative features were extracted and fed to train seven different types of machine learning algorithms. Trained machine learning algorithms were validated using leave-one-subject-out cross-validation. Support Vector Machine (SVM) algorithms were most successful, and could detect and classify stumbles with 100% sensitivity, 100% specificity, and 96.7% accuracy in the independent testing dataset. The SVM algorithms were implemented in a user-friendly, freely available, stumble detection app named Stumblemeter. This work shows that stumble detection and classification based on SVM is accurate and ready to apply in clinical practice.

## 1. Introduction

### 1.1. Stumbling in Individuals with Impaired Gait

Among non-disabled older adults, tripping over an obstacle has consistently been reported as the leading cause of falls [1,2,3], accounting for 33 [3] to 53 percent of all falls [2]. Fall risk is even increased in chronic disorders such as osteoarthritis [4], stroke [5], and leg amputees [6]. During gait, an individual may be particularly susceptible to tripping or stumbling at the instant when the swing foot reaches its peak forward velocity and, simultaneously, the vertical distance between the swing foot and the ground reaches a local minimum [7]. This point in the gait cycle has been referred to as the instant of minimum toe clearance (MTC). Theory predicts that small MTC and larger toe clearance variability increase the probability that the swing foot will contact an unseen obstacle, initiating a stumble [8]. In the absence of compensatory strategies, the lack of ankle dorsiflexion muscles for individuals with a prosthesis is expected to affect MTC, possibly increasing the likelihood of stumbling over an obstacle [9]. Measuring the number of stumbles during daily life could be an effective way to identify older adults and impaired individuals who are prone to fall. Fall risk is directly associated with stumbles [10,11]. Sgyrley et al. [12] found that the elderly who reported multiple near falls were more likely to fall prospectively. For amputees, osseointegration is an innovative way to anchor the prosthesis to the bone of the stump. Such a direct skeletal connection of the prosthesis is claimed to provide superior walking stability over traditional prostheses [13,14]. However, scientific evidence is required to support these claims. Screening for individual fall risk is advocated for groups at risk (e.g., people with osteoarthrosis [4] or amputees), to indicate and tailor fall-prevention interventions.

### 1.2. Automatic Stumble Detection for an Objective Evaluation of Fall Risk

Assessing the number of stumbles is often based on subjective self-reports [15]. Even though these self-reports are a low-cost solution, they are not very accurate and reliable, but seriously biased, due to denial and under- or overestimation of the true occurrence of the stumbling events [14,16]. Therefore, accurate and reliable methods for objective detection of stumbles are required. Automatic stumble detection would enable clinicians to objectively assess patients who are at fall risk or monitor how an older individual’s fall risk changes over time. Moreover, such a system could be used to evaluate the efficacy of interventions that aim to promote walking safety. Furthermore, it can be used to monitor patient progress during fall-prevention training programs. In addition to the number of stumbles, also identification of the type of stumble recovery strategy used could be important information to inform therapists. The body has two primary approaches to recovering from stumbles [17,18]. In the elevating strategy, the obstructed foot is lifted over the impeding object and swung quickly forward to take the weight. In the *lowering* strategy, the obstructed foot is put onto the ground to take the body weight while the other leg performs a quick recovery step.

### 1.3. Wearable Sensors and Machine Learning

In near fall detection research, the rapid development in sensor technology and improvement of data-processing capabilities of devices has led to a shift from self-reports to remote monitoring using wearable sensors and advanced detection algorithms, as it gives the opportunity to potentially collect data outside the laboratory setting [19]. Especially the combination of an accelerometer and a gyroscope, also known as an inertial measurement unit (IMU), has become more popular, as the development of micro-electro-mechanical systems (MEMS) technology has led to the low cost, low mass, and low energy consumption of sensors.

However, if a stumble detection system is to be used in a real-world environment, it is hard to distinguish peaks in acceleration and angular velocity that are caused by stumbles from peaks that are caused by other movements, such as walking down the stairs. Using a threshold-based algorithm leads to dilemma: if it is too low, the device will also detect negative events (“false positive”), but if the threshold is too high, it will not detect positive events (“false negative”). The threshold is also dependent on the subject-to-subject variability [20]. Threshold-based algorithms cannot overcome this difficulty and more advanced algorithms are required to separate stumbles from other movements. Machine learning involves the development of algorithms that would enable computers to learn complex patterns and make intelligent decisions based on these algorithms, without explicitly being programmed to do so [21]. The development of advanced machine learning algorithms offers the possibility to classify complex data. However, machine learning is still a relatively new field in stumble and fall detection research. Different techniques have been developed to automatically identify stumbles with varying degrees of success using body-fixed sensors [15,22,23,24,25,26,27,28]. Sensitivities and specificities ranged from 75 to 100% and 90.1 to 100%, respectively. However, only one study by Aziz et al. [22] included activities of daily living (ADLs) in their dataset. Activities of daily living should be added in the dataset to check the accuracy of the developed stumble detection system and its ability to differentiate between stumbles and ADLs. Although Aziz et al. achieved 100% sensitivity and 100% specificity, their system required five sensors. They achieved 92.0% sensitivity and 99.2% specificity with a single sensor placed on the right thigh. No study aimed to create a lower leg-based stumble detection system. A lower leg-based stumble detection system could be of value to individuals with a prosthesis, as it offers the possibility to attach the sensor to the prosthesis. Furthermore, no study aimed to create a system able to classify the type of stumble (elevating strategy or lowering strategy).

The goal of the present study is to investigate whether a single IMU sensor placed on the lower leg together with machine learning algorithms can be used to detect and classify stumbling events, with high sensitivity and specificity, in a context of activities of daily life.

## 2. Materials and Methods

### 2.1. Participants, Experimental Setup, and Protocol

Ten healthy volunteers (9 young (25.4 ± 1.5 years) and 1 older (60 years)) participated in the study. The study was approved by the TU Delft Human Research Ethical Committee (HREC-1304). All risks and precautions of the experiment were explained to the participants, after which they read and signed the informed consent form.

**Figure 1:**
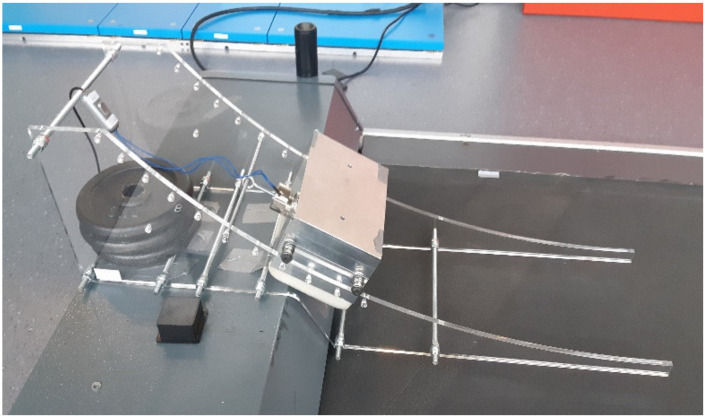
Stumbling device [29] mounted on a treadmill.

To make the participants stumble unexpectedly, a stumbling device based on the design by King et al. [29] was built. The device consists of a ramp-based obstacle delivery apparatus that releases an obstacle onto a treadmill (see Figure 1). The obstacle was made out of aluminum and weighted approximately 6 kg. The horizontal velocity at treadmill touchdown could be modified by changing the point along the ramp where the obstacle is held by an electromagnet. When the obstacle was released, it rolled down the ramped track, on a set of flanged roller bearings mounted on shoulder bolts threaded into each corner of the obstacle, and then slid onto the treadmill belt. Firm foam padding was attached to the front and bottom of the obstacle to protect the subjects’ toes and the treadmill belt, respectively.

**Figure 2:**
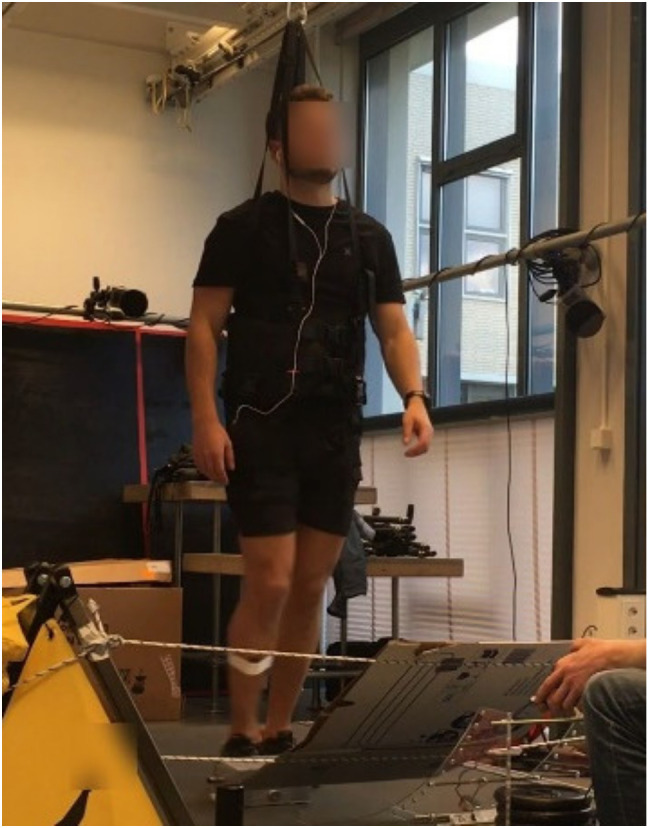
Stumbling device and experimental setup.

Participants were asked to walk steadily on a treadmill and manage the unexpected tripping perturbations. To prevent subjects from hearing or seeing the obstacle being deployed, each subject listened to music via earbuds, and a shield was placed directly above the stumbling device, to occlude visual perception of the obstacle sliding on the treadmill (see Figure 2). Participants wore a safety harness that was attached to the ceiling by a cord and a stiff spring, to prevent them from falling. Participants were given several minutes to walk on the treadmill before testing, to acclimate to the setup. During the stumbling trials, the treadmill speed was changed after every three consecutive stumbles, ranging from 1 to 5 km/h, to elicit different gradations of stumbles and to prevent habituation. Changes in treadmill speed were chosen randomly. Release of the obstacle on the treadmill happened about once every minute. The legs of the participants were videotaped, to classify a trial as either a successful stumbling trial or a mistrial. A trial was labelled as successful if there was a clear impact of the swinging foot with the obstacle during the swing phase. Trials were labelled as unsuccessful if the subject stepped on or over the obstacle. For each test subject, at least 20 successful stumbles were recorded. Stumbling trials were divided into two classes based on the recovery strategy used: the elevating strategy or the lowering strategy [30,31]. The experimenter ensured that about the same number of elevating stumbles and lowering stumbles were evoked by manually timing the release of the obstacle. The recovery strategy was determined by the trajectory of the perturbed foot after impact:

- Elevating strategy: After impact with the obstacle, the perturbed foot lifts up and over the obstacle, landing past the obstacle. This strategy is used when the foot is perturbed in the early swing phase (5–50% of the entire swing phase).
- Lowering strategy: After impact with the obstacle, the perturbed foot lowers in front of the obstacle, while the other foot performs a recovery step and lands past the obstacle. This strategy is used when the foot is perturbed in the late swing phase (40–75% of the entire swing).

**Figure 3:**
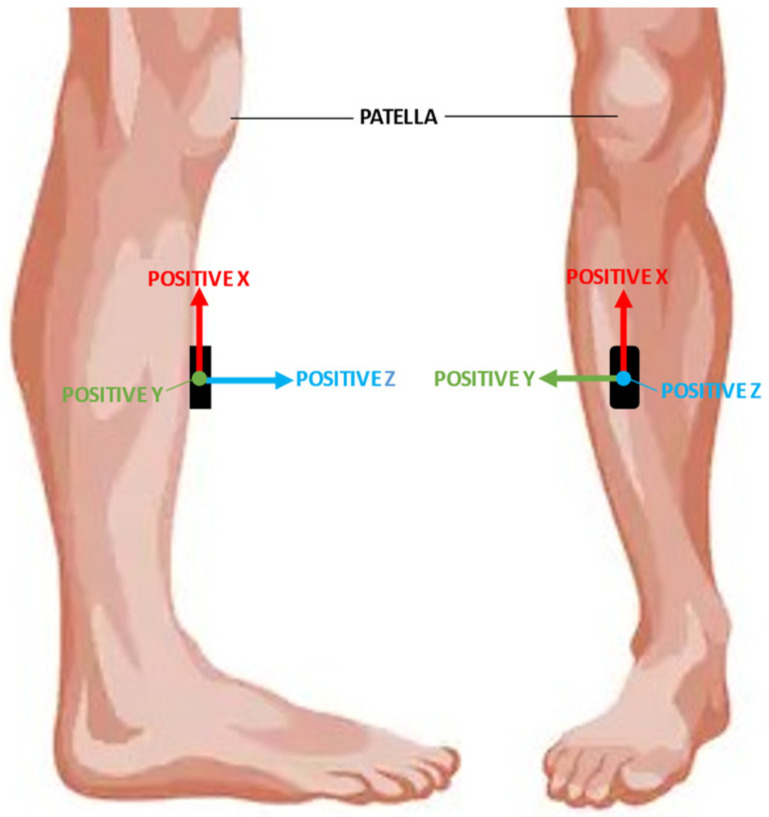
Placement and direction of the axes of the Ax6 sensor.

One Ax6 inertial measurement unit (IMU) from Axivity, Newcastle upon Tyne, UK, was used during this study. The IMU was placed on the tibia, 20 cm below the patella, using sports tape for fixation to the skin. The sensor was set to record at 100 Hz, with an accelerometer range of ±8 g and a gyroscope range of ±500 dps. The placement and directions of the axes of the IMU are shown in Figure 3.

After the stumbling trials, the participants performed several Activities of Daily Living (ADLs) that resemble common movements that are present in the daily life of individuals with a prosthesis. The inclusion of ADLs in the training dataset is necessary to properly train the machine learning classification models and reduce the number of false positives and false negatives when the system is used in the real world. Each ADL was explained and demonstrated to the participants by the experimenter. The participants then performed the ADLs themselves (see Table 1).

### 2.2. Dataset and Software

Accelerometer and gyroscope data collected during the experiments were uploaded to a computer via Omgui (Newcastle: Open Movement Newcastle University) version V1.0.0.43., an open-source lightweight application. Omgui is used to set up and configure the Axivity sensors, as well as to visualize the data.

The video recordings of the legs of the test subjects were synchronized with the IMU sensor data via ELAN (Nijmegen: Max Planck Institute for Psycholinguistics) version 5.9. In ELAN video recordings and IMU sensor data can be synchronized and played back. To obtain the ground truth, the experimenter used this application to manually label the different activities in MATLAB. Motions were labelled as ‘Stumble (Elevating)’, ‘Stumble (Lowering)’, and ‘Other’. Mistrials were labeled as ‘Other’ and kept in the dataset. The labelled activities (classes) from the video footage were treated as the ground truth, to train the machine learning models.

MATLAB (Mathworks Inc., Natick, MA, USA) release R2020b version 9.9.0.1592791 was used for processing the data and developing the machine learning models.

### 2.3. Data Pre-Processing

The dataset required minimal pre-processing. Data from the IMU contains seven columns. The first column contains the time as a serial date number. Columns 2 to 4 and 5 to 7 contain the accelerometer and gyroscope data in X, Y, and Z direction, respectively. The logging frequency was set to 100 Hz, resulting in 100 data points per second. The serial date numbers were converted to a datetime array using the MATLAB function *datetime*. The resultant acceleration without gravity (g) and resultant angular velocity (deg/s) at each time was calculated using Equations (1) and (2):

(1) $$a_{r}=\sqrt{a_{x}^{2}+a_{y}^{2}+a_{z}^{2}}-1$$

(2) $$\omega_{r}=\sqrt{\omega_{x}^{2}+\omega_{y}^{2}+\omega_{z}^{2}}$$

In total, 8 signals were used for machine learning; $a_{x},\;a_{y},\;a_{z},a_{r},\;\omega_{x},\;\omega_{y},\;\omega_{z},$ and $\omega_{r}$.

### 2.4. Window Segmentation and Labelling

In machine learning problems where time series come into play, the data should be adequately partitioned and labelled with the corresponding activity, to distinguish between the different classes. In human activity classification, several windowing techniques are used to divide the sensor data into smaller time segments (or windows), also known as window segmentation. Subsequently, feature extraction is applied to each window separately.

**Figure 4:**
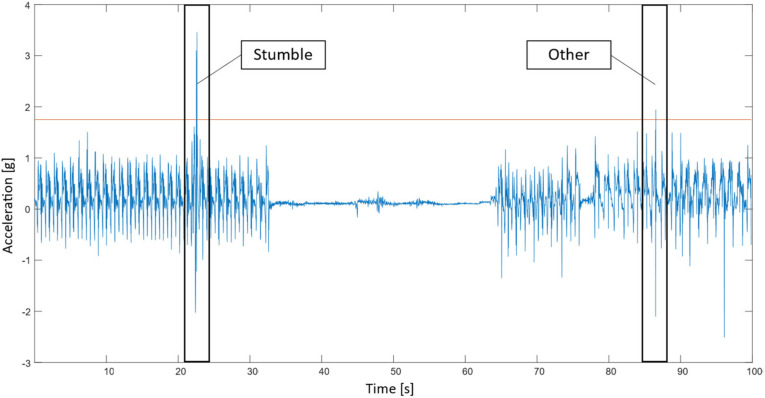
Event-defined window segmentation technique. Time windows were created around the intersection points between the accelerations in the (forward) z-direction (blue) and the threshold line (red).

In our approach, an event-defined window segmentation method was used. The first step in the event-defined window segmentation method is to find potential stumbles in the dataset. As each stumble is characterized by peaks in acceleration, the *findpeaks* option in MATLAB is used to locate peaks in the dataset. As both stumbles using an elevating strategy and lowering strategy are characterized by high acceleration peaks in the forward z direction, the *findpeaks* function in MATLAB was used to find peaks in this signal. To reduce the number of peaks considered, a threshold value of 1.75 g was empirically chosen, as this value was just low enough to capture all stumbling peaks, as well as some peaks caused by other movements. Furthermore, the time interval in-between peaks was set to 4 s, ignoring the lower peaks within this range. This ensures that stumbles are not detected multiple times, as the acceleration signal may cross the 1.75 g threshold multiple times during a stumble. The *findpeaks* option returns the locations (indices) of the peaks. After the locations of the peaks were found, these locations were used as the centers of the windows. Time windows of 2560 milliseconds were created as this length is enough to fully capture a stumble. For each location, 1270 milliseconds before the peak to 1280 milliseconds after the peak was considered to form a time window (see Figure 4). As the sampling frequency of the IMU was 100 Hz, each time window includes 256 data points for one signal. As there are 8 signals, each window contains 8 × 256 = 2048 data points. The event-defined window segmentation method reduces the computational time as only parts of the data that are potential peaks are fed into the machine learning algorithms and the rest of the data, the vast majority, is ignored for the rest of the process.

Next, the time windows were labelled. During this study we evaluated two different approaches to classify the data into three classes: *Stumble (elevating)*, *Stumble (lowering)* and *Other.* In the first approach, the three-class classification approach, we tested the capability of the different machine learning algorithms to directly classify the data into the three classes. We use dataset D1, where the data are grouped by the three classes, to evaluate this approach (see Table 2). In our second approach, the double binary classification approach, we tested the capability of the different algorithms to first classify the data into two classes: *Stumble* and *Other*. Subsequently, all windows predicted as stumbles were classified as either *Elevating* or *Lowering* using a second machine learning algorithm. We used datasets D2 and D3 to test this approach (see Table 2).

### 2.5. Feature Selection and Extraction

Feature selection is an important area in machine learning. It is the process of selecting the relevant features to construct a model. The main idea behind feature selection is that some features are redundant or irrelevant and can therefore be removed without much information loss. Research has shown that it is an effective way to improve the learning process and recognition accuracy and decreases the complexity and computational cost. Some models are negatively affected by irrelevant features [32]. The main objective of feature selection in supervised machine learning is to improve the classification accuracy and reduce complexity [33,34].

In this study, both time domain and frequency domain features were tested and selected. A Fast Fourier Transform was used to extract the frequency-domain features. Initially, 42 different feature classes were tested for usability. For each time window, a single feature class was extracted per IMU signal, creating 8-dimensional feature vectors (1 feature class × 8 signals). These feature vectors were then fed into different machine learning algorithms, to test the feature classes’ predictive power. Feature classes were only selected if they were capable of achieving at least 70% sensitivity and specificity with a machine learning algorithm, indicating there is a strong correlation with the output. A total of 16 features classes passed the first selection round (see Table 3).

Next, for each time window, all 16 features classes were extracted for each of the 8 IMU signals, creating 128-dimensional feature vectors (1 classes × 8 signals). This means that for each time window there are 128 features that could describe the characteristics of that window. An effective method to identify the most relevant features is sequential feature selection [35]. Sequential feature selection is a wrapper-type feature selection algorithm that starts training using a subset of features and then adds or removes a feature using a selection criterion. The selection criterion directly measures the change in model performance that results from adding or removing a feature. The algorithm repeats training and improving a model until its stopping criteria are satisfied. This method has two components:

- An objective function, called the *criterion*, in which the method seeks to minimize the overall feasible feature subsets. For our classification problem, the misclassification rate was set as the objective function.
- A sequential search algorithm, which adds or removes features from a candidate subject while evaluating the criterion.

The method has two variants:

- *Sequential forward selection* (SFS), in which features are sequentially added to an empty candidate set until the addition of further features does not decrease the criterion.
- *Sequential backward selection* (SBS), in which features are sequentially removed from a full candidate set until the removal of further features increase the criterion.

SFS was chosen over SBS for feature selection as the computational cost is significantly lower with SFS. For each model, features were selected using SFS with 30 objective evaluations.

Finally, we normalized the extracted features to rescale the data to a common scale. Supervised machine learning algorithms learn the relationship between the input and output and the unit, scale, and distribution of the input data may vary from feature to feature. This will impact the classification accuracy of the models. In this work, the data were normalized by scaling each input variable to a range of 0 to 1.

### 2.6. Machine Learning Algorithms

After the sensor data were properly processed and the features were extracted; the next step is to feed these feature vectors to a machine learning algorithm. In this study, seven types of machine learning algorithms were tested: Decision Tree [36], Discriminant Analysis [37], Logistic Regression [38], Naïve Bayes [39], Support Vector Machine (SVM) [40], k-nearest neighbors (KNN) [39], and Ensemble Learning [41]. Each type of machine learning algorithm has hyperparameters to select. For each type of machine learning algorithm, the optimal set of hyperparameters was found for the three different machine learning classification datasets, by using a Bayesian Optimization Algorithm with 40 iterations (see Appendix A for an overview of the Machine Learning algorithms that were trained and evaluated, with their optimal hyperparameters).

### 2.7. Training, Validating, and Testing

**Figure 5:**
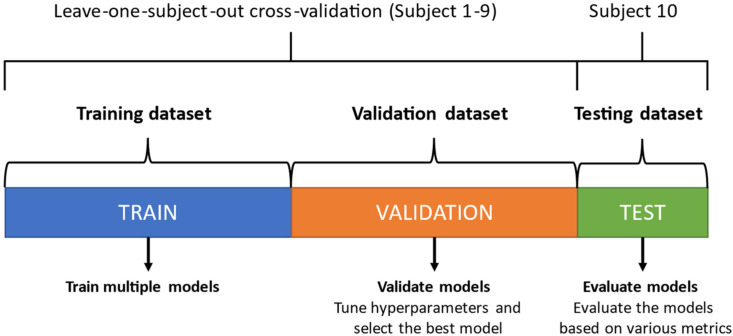
Division of the dataset into a training dataset, validation dataset, and testing dataset.

To evaluate the different machine learning algorithms, the dataset was divided into a training dataset, validation dataset, and testing dataset (see Figure 5). To determine the optimal hyperparameters, leave-one-subject-out cross-validation (LOOCV) was used together with Bayesian Optimization on the data from Subjects 1 to 9—the younger test subjects. Like k-fold cross-validation, the data were partitioned into training data and validation data. The validation dataset provides an evaluation of a model fit on the training dataset while tuning the model’s hyperparameters [42]. With 9 subjects, the cross-validation process iterated 9 times. For each iteration, the data of the left-out subject was used as validation data and the data of the remaining subjects as training data. After the 9 iterations, the predicted labels of the validation data were compared with the true labels. Trained models with the optimal hyperparameters, found using Bayesian Optimization, were exported.

The trained models were then evaluated with the testing data from the remaining Subject 10—the older test subject. The testing dataset is a dataset used to provide an unbiased evaluation of a final model fit on the training dataset [42]. This testing dataset was not used for training. The predicted labels of the testing data were compared with the true labels.

Next, the total performance for each model was calculated with different metrics. For this study, the most important performance metrics to compute were sensitivity (also called true positive rate, hit rate, or recall), specificity (also called true negative rate), and accuracy (see Equations (3)–(5)). These metrics were calculated after training and validating with LOOCV (validation scores), and after testing the exported models on the holdout data from Subject 10 (test scores). It should be noted that the specificities were calculated over the reduced dataset, containing just time windows with peaks that cross the threshold.

(3) $$Sensitivity=\frac{TP}{TP+FN}$$

(4) $$Specificity=\frac{TN}{TN+FP}$$

(5) $$Accuracy=\frac{TE+TL}{Total\;stumbles}$$

where *TP* represent the true positives (true stumbles), *TN* represents the true negatives (true ADLs), *FP* represents the false positives (ADLs misidentified as stumbles), and *FN* represents the false negatives (stumbles misidentified as ADLs). *TE* represent true elevating stumbles and *TL* the true lowering stumbles.

## 3. Results

In total, 276 successful stumbles were captured by the IMU, of which 134 were stumbles that were recovered using the elevating strategy and 132 that were recovered using the lowering strategy. Subject 10 stumbled 30 times and recovered from 5 perturbations by jumping over the obstacle with both legs at the same time. These ‘hopping’ recoveries were labelled as elevating, as the obstructed foot was lifted over the object directly after the collision. No separate class was created for these ‘hopping’ stumbles as there was simply not enough data to do so. The dataset of all subjects combined, including both the stumbling data and ADLs, is approximately 11.5 h long.

In this chapter, all the different machine learning algorithms are validated using leave-one-subject-out cross-validation and tested by using the exported models on the holdout data from Subject 10. In Section 3.1, single machine learning algorithms were used to separate three classes directly: *Stumble (elevating)*, *Stumble (lowering)*, and *Other.* In Section 3.2, two machine learning algorithms were used in series, to first separate all stumbles from all other peaks, and subsequently differentiate between the type of stumble recovery strategy. Sensitivity and specificity were computed to validate and evaluate the model’s ability to separate the stumbles from the other data. Accuracy was computed to evaluate the model’s ability to distinguish between stumbles where an elevating strategy was used and stumbles where a lowering strategy was used.

### 3.1. Three-Class Classification Approach

**Figure 6:**
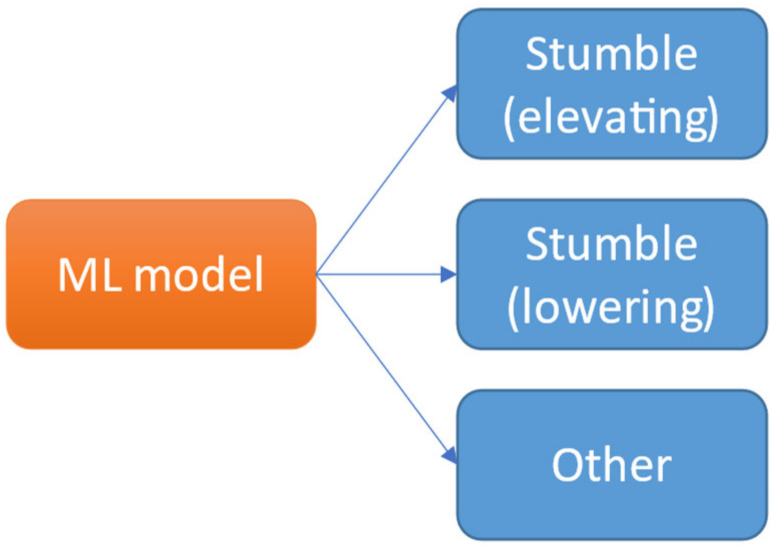
Single machine learning model to classify data into three classes.

Since we do not only want to separate stumbles from the other data, but also want to distinguish between the type of stumble (elevating strategy/lowering strategy), there are three classes in this machine learning classification problem. Intuitively, a single machine learning model can be used to classify the data into the three classes (see Figure 6).

Table 4 and Table 5 show the results for this classification problem. We used dataset D1 to validate and evaluate this approach. The model predictions were compared with the true labels. Sensitivities and specificities were calculated by taking both types of stumbles together as the positive class and the ‘Other’ windows as the negative class. Accuracy was calculated by the number of correctly classified stumbles (elevating as elevating and lowering as lowering) divided by the number of detected stumbles. The highest sensitivities, specificities, and accuracies where achieved with the SVM model during both validation and testing.

### 3.2. Double Binary Classification Approach

**Figure 7:**
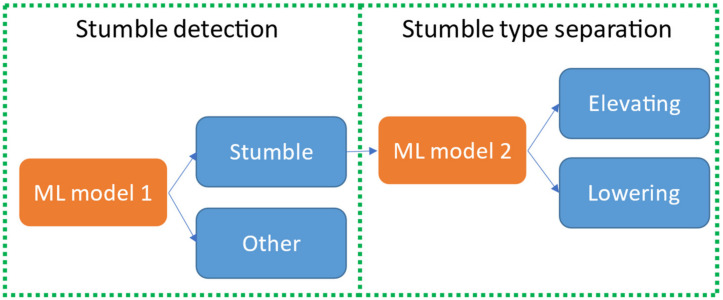
Two binary machine learning models in series.

In our second approach, the machine learning classification problem was split into two parts. First, a machine learning model was used to detect stumbles in the data. We call this the stumble detection problem. Subsequently, a second machine learning model was used to classify the stumbles as either a stumble where an elevating strategy was used or a stumble where a lowering strategy was used. We will call this the stumble type classification problem (see Figure 7).

Table 6 and Table 7 show the results for the stumble detection problem, validated and evaluated with dataset D2. The model predictions were compared with the true labels. A confusion matrix was created for each model and the sensitivities and specificities were calculated for both validation and testing. The best results were achieved with the SVM model, with a 100% sensitivity and 100% specificity in the testing dataset. Table 8 and Table 9 show the results for the stumble-type classification problem, validated and evaluated with dataset D3. The accuracy was defined as the amount of correct predictions over the total amount of predictions. Again the SVM model outperformed other models, with an accuracy of 96.7% in the testing dataset.

### 3.3. Final Model

For our final model, we look at the results of the previous two paragraphs. As precise detection of the stumbles is prioritized over accurate stumble-type classification, the main demand for the final model is its ability to detect as many stumbles as possible with keeping the number of false positives as low as possible. For both validation and testing, the highest sensitivity and specificity were achieved with the double binary classification approach. For both the stumble detection problem and the stumble-type classification problem, the best results were achieved with SVM. For stumble detection, SVM achieved 100% sensitivity and 100% specificity in the testing dataset. For the stumble-type classification, the SVM was able to classify the stumble recovery type with 96.7% accuracy in the testing dataset. Therefore, for our final model we used these two SVM models in series. Table 10 shows the selected features using SFS and the selected hyperparameters using Bayesian Optimization for both models.

### 3.4. Stumblemeter App

**Figure 8:**
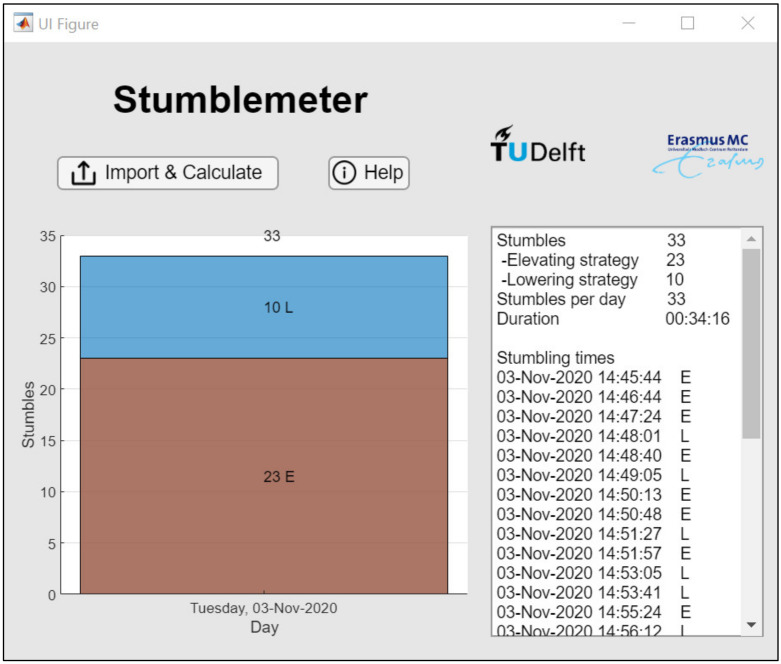
Interface of the Stumblemeter app. The number of stumbles per day is displayed on the left in the form of a histogram. The total number of stumbles, the duration of the measurement, and the individual stumbling times are displayed on the right.

To make the programming work of this study accessible for clinicians, an application named Stumblemeter was created (see Supplementary Materials). After uploading the .cwa file containing the IMU data, the application automatically performs all the steps required for machine learning classification (see Figure 8 for the interface of the Stumblemeter app). The application displays the number of stumbles in the form of a histogram. In the text area, the total amount of stumbles during a measurement is displayed, as well as the times when a stumble occurred. Depending on the physical activity of an individual with a prosthesis, the computation time for a 7-day measurement is about 3 min.

## 4. Discussion

### 4.1. Recap

During this study, seven types of machine learning algorithms were trained, validated, and tested. For each type, optimized hyperparameters were found using Bayesian Optimization. All the models were first validated using leave-one-subject-out cross-validation, and then exported. The exported models were then tested on the testing dataset, which included the data from the older test subject. We found that using two binary SVM models in series produced better results that using a single SVM to directly classify the data into three classes. Therefore, these two SVMs were used in the final model. Even though the subjects performed a multitude of different ADLs, no other movements were recognized as a stumble in both the validation dataset and the testing dataset.

### 4.2. Internal Validity

The data were split into a training dataset, validation dataset, and testing dataset. The validation dataset is a sample of data held back from training the model that is used to give an estimate of the model skill while tuning the model’s hyperparameters. The validation dataset is different from the test dataset, which is also held back from the training of the model, but is instead used to give an unbiased estimate of the skill of the final tuned model when comparing or selecting between final models [42].

For each type of machine learning algorithm, Bayesian Optimization was used during leave-one-subject-out cross-validation with data from nine subjects to find the optimal set of hyperparameters. Models were trained with Bayesian Optimization to minimize the error function defined with respect to the training dataset. The performance of the models was compared by evaluating the error function using an independent validation dataset, which provided an evaluation of a model fit on the training dataset while tuning the model’s hyperparameters, and the models having the smallest error with respect to the validation dataset were selected. Since this procedure can itself lead to some overfitting to the validation dataset, the performance of the selected models was confirmed by measuring its performance on a third independent test dataset, containing the data from Subject 10. Testing with the unseen data provided an unbiased evaluation of the final model fit of the training dataset [42]. The predicted labels were compared with the true labels and the sensitivity, specificity, and accuracy of the models were calculated to evaluate the models. By using this method, it is certain that the testing data could not have influenced the training of the models.

### 4.3. Comparison with Previous Studies

In previous near-fall detection research, only two studies achieved 100% specificity. Aziz et al. [22] did include multiple ADLs in their dataset. However, the way they recreated stumbles is questionable, as they had their participants act out a stumble on a mattress after watching a video. It remains unclear whether their system would be able to accurately detect a real-world stumble. Moreover, their setup is too impractical for clinical use: it consists of five sensors of which one was placed on the head. The other study that achieved 100% specificity, by Choi et al. [23], added just three ADLs in their dataset—standing, walking, and lying down—and did not include activities with high acceleration peaks. Such a limited dataset lacks realistic representation of real-life activity, which could result in an overestimation of the practical performance. Two sensors were used, which are less attractive for practical use than our single-sensor system.

All in all, we expect that the Stumblemeter presented in this study will outperform previously reported systems. Importantly, the machine learning algorithm was trained and tested with naturally occurring stumbles in a dataset that contains a representative number of ADLs. For clinical feasibility, it is important that the single sensor can be attached to the shank in an unobtrusive way and can be worn for a longer period of time, e.g., a week.

This study also aimed to create an algorithm that is able to determine the type of stumble, whether an elevating recovery strategy was used or a lowering recovery strategy. A second model was used to classify the detected stumbles into the two classes. This model was trained, validated, and tested, separately. We found that an optimized SVM was able to distinguish between the two types of strategies with 96.7% accuracy in the testing dataset.

In terms of computational cost, we cannot compare our system with other systems [15,22,23,24,25,26,27,28] as they did not give any specifications on that matter. However, it is evident that the event-defined window segmentation technique that was introduced ensures that the computational cost is considerably lower than when the full dataset has to be processed. We made use of the fact that all stumbles are paired with high peaks in acceleration. By using a threshold, the vast majority of irrelevant data (95.5% in our dataset) is eliminated at an early stage. As a result, a limited amount of time-window features have to be extracted and fed into the machine learning models. In this study, 682 high-peak time windows that were created, of which 276 (40.5%) were stumbles and 406 (59.5%) were non stumbles. The estimated computational time for a week-long measurement is 3 min at most, depending on the user activity.

### 4.4. Practical Application in Clinical Research

The Stumblemeter has been validated and tested in healthy people. Strictly spoken, this will not guarantee that it will work as well in the target population: individuals with walking difficulties, e.g., those walking with a prosthesis. The system would have to be tested separately for the different pathologies. Nonetheless, it is expected that the Stumblemeter will work as desired during clinical research, e.g., in the amputee population. Shirota et al. [43] showed that transfemoral amputees generally exhibited typical able-bodied recovery strategies (elevating and lowering) when recovering from stumbles on both the sound and prosthesis sides. They found that throughout the swing phase, amputees used similar recovery strategies to able-bodied subjects for perturbations that occurred at similar time points in the gait cycle. However, two out of eight amputees in their study used a novel hopping strategy when tripped using a tether on the prosthesis side in early to mid-swing. This strategy was also found in the older test subject in our study. Such recoveries were labelled as elevating, as the obstructed foot was lifted over the object directly after the collision. These ‘hopping’ stumbles were classified as elevating stumbles and were all detected correctly. Therefore, it is expected that our system is able to detect such stumbles, even though it is not specifically trained to classify this particular recovery type. Follow-up research on individuals with prosthetic legs should be conducted to validate this expectation.

## 5. Conclusions

This work shows that stumble detection and classification based on an IMU and SVM is extremely accurate and ready to apply in clinical practice. Our proposed system consists of just one small IMU sensor, which can easily be integrated into the pylon of a prosthesis or attached to the shank, leaving no burden for the users. Out of the 30 evoked stumbles from an independent experiment, the optimized SVM model was able to detect all of them (100% sensitivity). Moreover, our models did not give any false-positive predictions (100% specificity), even though the dataset comprised of a wide variety of daily movements. Moreover, this is the first study aiming to classify the type of stumble recovery strategy, which it did with 96.7% accuracy. The user-friendly Stumblemeter app makes it quite straightforward for clinicians to analyze the data. The introduction of the Stumblemeter enables clinicians to objectively assess fall risk in older adults, amputees, and other individuals with gait impairments, outside a laboratory or clinical setting.

## Figures and Tables

**Table 1:** Activities of Daily Living.

ADL	Amount/Time	Instructions
Walking straight	5 min	1, 2, 3, 4, and 5 km/h on a treadmill (1 min each)
Walking corner	10×	Walk 90-degree and 180-degree corners
Come to a halt	10×	Stand still after walking. Repeat 10 times.
Sitting and rising	10×	Sit down on a chair and rise from a chair in different ways and speeds. Repeat 10 times
Pick up object from ground	10×	Throw a small ball on the ground and then pick it up from the ground in different ways and speeds. Repeat 10 times.
Walking upstairs and downstairs	5×	Walk up and downstairs in different ways and using speeds. Repeat 5 times

**Table 2:** Labelled datasets.

Dataset	Classes	Amount of Windows (Validation)	Amount of Windows (Test)
(D1) Three-class classification	Stumble (elevating) Stumble (lowering) Other	132 114 329	12 18 77
(D2) Stumble detection	Stumble Other	246 329	30 77
(D3) Stumble type classification	Elevating Lowering	132 114	12 18

**Table 3:** Feature classes.

Nr	Feature Class
1	Interquartile range
2	Kurtosis
3	Mean
4	Median
5	Mean absolute deviation
6	Maximum
7	Minimum
8	Peak-magnitude-to-RMS-ratio
9	Spectral entropy
10	Prominence
11	Root-mean-square level
12	Root-sum-of-squares level
13	Range
14	Skewness
15	Standard deviation
16	Sum of local maxima and minima

**Table 4:** Results of the three-class classification approach (validation).

ML Model	Sensitivity (%)	Specificity (%)	Accuracy (%)
SVM	98.4	99.4	98.5
Ensemble Learning	98.0	98.5	93.4
Discriminant Analysis	97.2	97.0	90.0
KNN	97.2	95.1	74.1
Naïve Bayes	91.1	95.4	75.4
Decision Tree	87.8	93.3	77.8

**Table 5:** Results of the three-class classification approach (testing).

ML Model	Sensitivity (%)	Specificity (%)	Accuracy (%)
SVM	96.7	100	96.6
Ensemble Learning	93.3	98.7	92.9
Discriminant Analysis	93.3	97.4	89.3
KNN	90	93.5	88.9
Naïve Bayes	90	93.5	77.8
Decision Tree	83.3	94.8	72.0

**Table 6:** Results of the stumble detection problem (validation).

ML Model 1	Sensitivity (%) (Validation)	Specificity (%) (Validation)
SVM	98.8	100
Discriminant Analysis	98.0	96.3
Ensemble Learner	97.6	98.2
Logistic Regression	97.6	94.8
KNN	96.3	92.4
Naïve Bayes	88.2	90.4
Decision Tree	88.0	93.6

**Table 7:** Results of the stumble detection problem (testing).

ML Model 1	Sensitivity (Testing)	Specificity (%) (Testing)
SVM	100	100
Discriminant Analysis	96.7	97.4
Ensemble Learner	96.7	97.4
Logistic Regression	90.0	93.5
KNN	90.0	92.2
Naïve Bayes	86.7	89.6
Decision Tree	83.3	88.3

**Table 8:** Results of the stumble-type classification problem (validation).

ML Model 2	Accuracy (%) (Validation)
SVM	95.5
Ensemble Learner	91.9
Discriminant Analysis	87.0
KNN	86.6
Logistic Regression	85.4
Naïve Bayes	84.2
Decision Tree	81.3

**Table 9:** Results of the stumble-type classification problem (testing).

ML Model 2	Accuracy (%) (Testing)
SVM	96.7
Ensemble Learner	93.3
Discriminant Analysis	83.3
KNN	80.0
Logistic Regression	80.0
Naïve Bayes	76.7
Decision Tree	73.3

**Table 10:** Final models: features and hyperparameters.

ML Model	Type	Features	Kernel Function	Box Constraint Level	Kernel Scale
ML model 1 (stumble vs. other)	SVM	Median $a_{x}$ Maximum $a_{x}$ Minimum $a_{z}$ Minimum $\omega_{y}$ Spectral entropy $\omega_{z}$ Peak-magnitude-to-RMS ratio $\omega_{z}$ Peak-magnitude-to-RMS ratio $\omega_{r}$	Linear	976.7	7.5492
ML model 2 (elevating vs. lowering)	SVM	Interquartile range $a_{r}$ Kurtosis $a_{x}$ Mean $a_{x}$ Mean $\omega_{x}$ Maximum $\omega_{z}$ Minimum $\omega_{r}$ Skewness $\omega_{r}$	Linear	0.0001	0.0293

## Data Availability

The data generated in this study are openly available in 4TU.ResearchData at https://doi.org/10.4121/14473320.

## Supplementary Materials

The following are available online at https://doi.org/10.4121/14473269: Stumblemeter app and MATLAB code.

## Appendix A

**Table A1:** Decision Tree: optimized hyperparameters for each dataset.

ML Model	Maximum Number of Splits	Split Criterion	Surrogate Decision Splits
Decision Tree (D1)	59	Maximum deviance reduction	Off
Decision Tree (D2)	38	Gini’s diversity index	Off
Decision Tree (D3)	19	Gini’s diversity index	Off

**Table A2:** Discriminant Analysis: optimal hyperparameters for each dataset.

ML Model	Discriminant Type
Discriminant Analysis (D1)	Linear
Discriminant Analysis (D2)	Linear
Discriminant Analysis (D3)	Linear

**Table A3:** Logistic Regression.

ML Model
Logistic Regression (D2)
Logistic Regression (D2)

Note 1: Logistic Regression can only be used for binary classification problems. Note 2: Hyperparameter optimization does not apply to Logistic Regression as there are no hyperparameters to alter.

**Table A4:** Naïve Bayes: optimized hyperparameters for each dataset.

ML Model	Distribution Type	Kernel Type	Support
Naïve Bayes (D1)	Kernel	Gaussian	Unbounded
Naïve Bayes (D2)	Kernel	Triangle	Unbounded
Naïve Bayes (D3)	Kernel	Gaussian	Unbounded

**Table A5:** Support Vector Machine: optimized hyperparameters for each dataset.

ML Model	Kernel Function	Box Constraint Level	Kernel Scale
SVM (D1)	Linear	0.0010	0.0025
SVM (D2)	Linear	976.7	7.5492
SVM (D3)	Linear	0.0001	0.0293

**Table A6:** K-Nearest Neighbors: optimized hyperparameters for each dataset.

ML Model	Number of Neighbors	Distance Metric	Distance Weight
KNN (D1)	1	Cosine	Squared inverse
KNN (D2)	5	Spearman	Squared inverse
KNN (D3)	1	Correlation	Inverse

**Table A7:** Ensemble Learner: optimized hyperparameters for each dataset.

ML Model	Ensemble Method	Learner Type	Max Number of Splits	Number of Learners	Learning Rate	Subspace Dimension
Ensemble Learner (D1)	Bag	Decision Tree	320	12	-	53
Ensemble Learner (D2)	GentleBoost	Decision Tree	5	485	0.001864	38
Ensemble Learner (D3)	Bag	Decision Tree	182	27	-	35
